# Implementing clinical practice guidelines into action: a qualitative study of managing knowledge translation in primary care organisations

**DOI:** 10.1186/s12961-025-01402-z

**Published:** 2025-10-14

**Authors:** Anna-Aurora Kork, Miia Marttinen, Harri Laihonen, Johanna Ruusuvuori, Juha E. Ahonen, Eila Kankaanpää

**Affiliations:** 1 School of Management, University of Vaasa, Vaasa, Finland; 2 Tampere University, Tampere, Finland; 3 University of Eastern Finland, Kuopio, Finland; 4 The Finnish Medical Society Duodecim, Helsinki, Finland

**Keywords:** Management, Clinical practice guideline, Evidence-based practice, Primary care, Qualitative research

## Abstract

**Background:**

Clinical practice guidelines (CPGs) are essential for enhancing healthcare quality and informing evidence-based clinical practices. Despite the availability of strategies, their implementation remains challenging due to the complexities of managing translation CPGs into practice, such as barriers to change, resource limitations and high costs. This study examines management mechanisms that offer valuable insights into how healthcare organizations can manage CPG implementation at the organizational level to optimise high-quality care.

**Methods:**

This qualitative study examines the management of CPG implementation using interview data (*n* = 33) from healthcare managers and clinicians in Finnish public primary care. The data were collected through seven focus group interviews across nine public primary care organizations. The interview transcripts were analysed using thematic analysis with a multidisciplinary approach.

**Results:**

CPGs are considered important tools for improving care quality and promoting shared evidence-based practices. The obstacles to managing implementation included dissemination difficulties, motivation challenges and information overload. Managers and clinicians had contrary views on their roles and responsibilities in CPG implementation. To lead the knowledge translation processes, managers emphasised unit managers’ support, dissemination and communication channels, whereas clinicians viewed CPG implementation as a grassroots effort and the responsibility of each individual. The results illustrate the need for enhancing shared views on CPGs and managing social implementation activities within organizations.

**Conclusions:**

Successful CPG implementation requires active managerial efforts and clinician dialogue to transform new evidence into locally viable practices. To inform more effective knowledge translation, the five identified management mechanisms included instructions; accountability structures; motivation, goal setting and feedback; communication strategies and participatory practices. In managing CPG implementation, a focus on interaction processes, motivation and feedback is essential for ensuring collective knowledge formation. This study improves the understanding of critical organizational knowledge translation processes by drawing attention to the previously underrepresented managerial aspects in CPG implementation studies. Future researchers, guideline developers, and policymakers should integrate managerial activities and clinician engagement in CPG implementation to ensure effective practices and healthcare quality.

**Supplementary Information:**

The online version contains supplementary material available at 10.1186/s12961-025-01402-z.

## Background

Clinical practice guidelines (CPGs) provide evidence-based recommendations to assist clinicians in making decisions and diagnosing and treating medical conditions to ensure high-quality care. Guidelines summarise current medical evidence to inform clinical practices about the benefits and harms of care interventions [1, 2].

Numerous studies on implementation strategies [3–5] have recognised the failure to translate research into practice [6, 7]. CPGs require active implementation efforts, leadership and engagement to be used effectively. The complexities involved in translating CPGs into shared clinical practices have been extensively addressed in implementation science [8]. Many complexities in CPG implementation are known to be related to organizational culture, climate or leadership [9–11]. These issues stem from poor management, such as a lack of teamwork or resources, unclear roles or insufficient knowledge in implementation [7]. Nevertheless, the managerial aspect of CPG implementation has largely remained implicit despite its potential to provide valuable insights into the implementation. Understanding mechanisms – those organizational determinants, key leadership elements and implementation drivers [12–14] – that explain why strategies fail and offer important lessons for effectively translating CPGs into practice.

This study investigates the management mechanisms that advance the implementation of CPGs within primary care by focusing on how knowledge translation is led in healthcare organizations to ensure high-quality care. While previous studies have examined CPG implementation in specific clinical areas, there is less research on the organizational-level processes that support implementation and clinical decision-making [5]. Most studies focus on key determinants of CPG implementation with locally tailored recommendations for effective dissemination [15, 16], such as contextual barriers or effective strategies [7, 17, 18], or organizational barriers to change [19]. The knowledge translation literature proposes various models and stages, from linear knowledge transfer to collaborative translation, to facilitate implementation processes [20–22].

Recently, attention has shifted toward managers’ roles in implementing evidence-based practices, especially in primary care [23, 24], nursing [12] and acute care settings [3]. While managers may act in various linkage agent roles supporting guideline dissemination, stakeholder engagement, and capacity building through education and feedback, their impact on CPG implementation has been inconsistent [25, 26]. Middle managers promote knowledge translation at the operative level by motivating staff, shared learning and adaptive leadership [3, 26] whereas top managers strengthen commitment and focus on strategic implementation policies [10, 27]. Nevertheless, management practices often remain non-formalised processes lacking professionals’ experience in implementation [12, 23, 28].

Critical managerial aspects, such as the role of leadership [14, 29], managerial knowledge brokering [26, 30] and professional practices [17, 31], are known as factors supporting implementation. Although CPGs aim to ensure care quality and clinical outcomes [32], the managerial perspective has been limited in addressing the challenges of knowledge translation [5, 22, 33]. While implementation leadership has gained attention, the specific mechanisms through which managers support CPG implementation remain underexplored [14, 16]. Evidence shows that managers play a pivotal role in translating CPGs into practice, especially middle managers who act as change agents by disseminating and synthesizing information, mediating strategy to daily activities and selling implementation [13, 16]. However, managers often experience limited resources, weak monitoring and low professional engagement [31]. These challenges stem from organizational constraints hindering implementation. Awareness of these obstacles is essential for strengthening implementation leadership [12]. Five complementary management strategies – managers’ feedback, ongoing interventions, monitoring, partnerships and team engagement [4] – indicate the close link between implementation processes and managerial practices.

This study aims to find out how primary care organizations manage CPG implementation to optimise high-quality care. We will focus on two organizational levels: the management and the clinical work to study how managers and clinicians describe and conceptualise existing organizational structures, processes and managerial practices aimed at facilitating the knowledge translation necessary for successful CPG implementation. By introducing management mechanisms to support CPG implementation, the study deepens understanding of the know–do gap in healthcare organizations [34, 35]

## Methods

We conducted a descriptive qualitative study using focus group interviews and employed reflexive thematic analysis [36] to explore two questions: (1) which kind of management mechanisms (that is, managerial practices and knowledge translation processes) are described to guide CPG implementation within Finnish public primary care organizations, and (2) what are the perceived obstacles – organizational or managerial factors – to managing CPG implementation in these organizations studied. Guided by these analytical questions, our aim was to uncover the management mechanisms through which knowledge translation is assumed to operate. This inductive study design enabled a context-sensitive analysis without presuming organizational uniformity or success. Adopting an interpretivist stance, we did not treat the interviews as direct representations of organizational reality. Instead, the themes are researcher-constructed abstractions, where managerial mechanisms were interpreted through the causal logics evident in participants’ talk and reflected through each researcher’s disciplinary lens in management, economics and social psychology.

### Data collection

We utilised two primary datasets (Table 1) collected as part of broader research project examining shared decision-making to advance CPG development. Dataset 1 comprised two online focus group interviews (2022) with managers from two public primary care organizations. Dataset 2 consisted of five in-person focus groups (2021–2022) with clinicians from nine public primary care organizations, ensuring both organizational and geographical diversity. The participants were recruited by research collaborators to include both administrative managers and physician leaders, excluding those at highest executive governance level. Of the 15 managers invited, 11 participated. Clinician invitations were distributed via the GP association for its active members.

**Table 1:** Description of datasets

Dataset	Number of focus groups, participants (range) and duration	Positions	Interview topics
#1 Managers	Two focus groups, Total of 11 participants (4 + 7 members/group) Duration: 51–61 min	Health service area managers, medical directors, chief physicians and deputy chief physicians	CPG implementation: decision-making structures and processes; types of information and data used
#2 Clinicians	Five focus groups, Total 22 participants (3 to 8 members in all groups) Duration: 67–102 min	General practitioners in primary care	Evidence-based clinical practices: decision-making regarding changes made in clinical practice; effects on clinicians’ treatment decisions; de-implementation of low-value care

All participants worked in Finnish public primary care, which is characterized by multi-professional, team-based practice. Healthcare centres provide wide range of services, such as health counselling, maternity clinics, school healthcare, dental care, first aid, appointments and inpatient care. Managers typically oversee multiple physician teams, wards or service units, often in close cooperation with head nurses. Clinicians are physicians responsible for treatments and community health. CPG implementation is shaped not only by formal managerial and clinical practices but also by interprofessional collaboration with nurses, physiotherapists, psychologists and social workers.

Manager interviews were conducted jointly by two authors (Laihonen and Kankaanpää), while clinician interviews were conducted individually by two researchers (Marttinen and Ahonen) one of with extensive clinical experience and CPG implementation. None of the researchers had prior relationships with the participants. All interviews followed semi-structured guides; the manager guide was piloted with minor refinements. Interviews were conducted in Finnish, either via Microsoft Teams or in organizational meeting rooms. All interviews were audio- or video-recorded, supplemented with field notes and transcribed verbatim. Excerpts were pseudonymised to prevent identification. Each focus group met once, and no member checking was undertaken. All participants provided informed consent – oral for managers, written for clinicians. The study was approved by the Ethics Committee of the Tampere Region (ID 17/2021).

### Data analysis

**Fig. 1:**
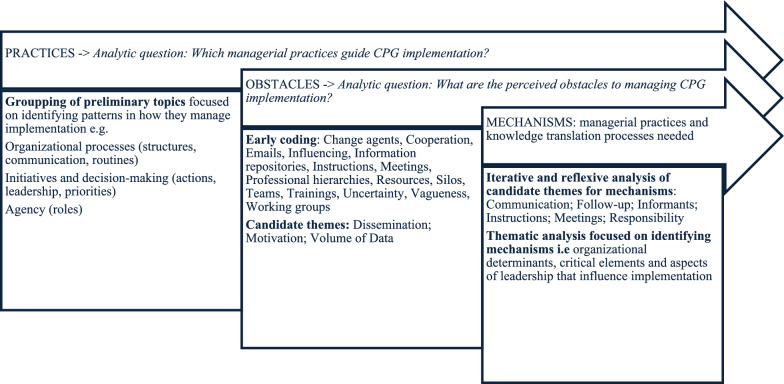
Phases of analysis

We analysed the data informed by Braun and Clarke’s [36] reflexive thematic approach in an iterative, context-sensitive manner (Fig. 1). Two researchers independently familiarized themselves with the raw interview data, grouping and highlighting extracts on CPG implementation, managerial practices and organizational knowledge translation. These preliminary topics and early codings, managed in Atlas.ti and Excel, served as entry points for subsequent phases of analysis, with memos maintained throughout.

Initial themes were generated by clustering patterned meanings, first focusing on managerial practices and then on perceived obstacles. Their coherence was repeatedly reviewed and discussed in research team meetings, with original passages revisited to preserve contextual nuance. Through several rounds, themes were merged, narrowed or discarded, then named and illustrated with extracts by all authors. To define the management mechanisms, we carefully reviewed each theme and analysed meanings attributed to those organizational determinants, critical elements, and aspects of leadership that influence implementation.

As a result, the final themes concerning obstacles (dissemination, information overload, and motivational challenges) and five management mechanisms (instructions and process descriptions, accountability structures, motivation, goal setting and feedback, communication strategies, and participatory practices) are presented as researcher-led abstractions rather than realist entities. Adequacy was judged in relation to study aims and contextual specificity rather than statistical saturation; the relatively small dataset enabled close attention to context while still identifying cross-cutting patterns.

### Reflexivity

We enhanced transparency and credibility through reflexive practices and researcher triangulation throughout the process [36]. Our multidisciplinary team bringing expertise in health management, health economics, knowledge management, social psychology and medicine enabled multiple perspectives and critical engagement with the data. We reflected on candidate themes and mechanisms in relation to disciplinary perspectives on knowledge construction, recognising that our backgrounds inevitably shaped what we attended to as meaningful. Divergent views were reconciled through extensive discussions and iterative abstractions. While this dialogue, spanning micro-, meso- and macro-level emphases, enriched our analysis, it also meant that alternative thematic accounts would have been possible.

## RESULTS: managing the translation of CPGs in primary care

### Obstacles to managing CPG implementation

Our results summarise two key dimensions of managing CPG implementation in healthcare organizations: the obstacles described by the interviewees and the managerial practices deployed to lead CPG implementation. Managers and clinicians expressed two divergent views on who is responsible for managing the implementation processes through which CPGs are translated into clinical practice. On the one hand, the interviewees framed implementation as grassroots efforts carried out by individual clinicians. On the other hand, collective action within the organization was deemed necessary.

This dual understanding was also reflected in how the interviewees perceived their own roles. Managers primarily saw themselves as decision-makers, emphasising the responsibility of unit managers and clinicians in the actual implementation of CPGs while also acknowledging their own organizational role in supporting shared evidence-based practices. Clinicians, in turn, valued collective processes in developing clinical practices but simultaneously viewed familiarising with CPGs as their individual responsibility.

Both managers and clinicians identified several obstacles to managing CPG implementation (Table 2). Managers frequently highlighted failed dissemination efforts, as desired changes in practice were not achieved despite communicating new CPGs to clinicians. Clinicians echo these concerns, describing an overwhelming volume of emails and instructions that were difficult to manage and absorb. Once filed in databanks, these documents were described as hard to locate and easily forgotten. Clinicians consistently emphasised challenges related to accessing guidelines, finding instructions and dealing with the flood of messages and Teams meetings regarding their implementation. Some interviewees acknowledged the difficulty of reaching everyone due to the large number of clinicians and many separate physical units.

A significant management obstacle identified by clinicians was a lack of motivation to implement new CPGs, which was attributed to insufficient support from organizational processes and managerial practices. This lack of incentive was owing to their inadequate engagement in the implementation process and limited interaction opportunities with management. Clinicians expressed a desire to be involved in designing changes in clinical practice and expected managers to provide the rationale for these changes. This would help staff members understand instructions and be more motivated to implement CPGs. Clinicians felt that management did not provide the necessary data, follow-up or evidence to justify and sustain the changes.

**Table 2:** The main obstacles in managing the translation of CPGs into practice

Examples of codes	Identified obstacles	Quote
Meetings Trainings Instructions Working groups Teams Change agents	**Dissemination difficulties:** Distant decision-making, “laissez-faire” attitude of implementation Knowledge translation as mere information delivery Unclear responsibilities and conflicting role expectations Time constraints in everyday work	*“At health stations, ward nurses and chief physicians are primarily responsible for putting CPGs into practice. We managers don’t implement these changes directly, our role is mainly to monitor progress, provide advice and guidance. Not actual implementation among the staff.” FG6_M6* *“This topic of implementation comes up frequently in the development working group. We discuss how to ensure that guidelines uploaded to the cloud service can be found. However, the real challenge lies in how effectively they are used, if users aren’t aware of their existence”* FG1_C2 *”…staff don’t have enough time to search for guidelines or read emails. Unless the guidelines are provided in a very accessible way, such as someone telling you at convenient time” FG4_C4*
Emails Information repositories Resources	**Information overload:** Underdeveloped feedback culture Poor sharing and utilisation of information Extent and complexity of information needed in clinical practice Lack of resources in development work	*“There is certainly no monitoring… Yeah, there are no – resources for that, and this will make it difficult. It feels like the grassroots actors who have been planning the change in operational level, and are implementing it, should receive follow-up information. If the results and impacts are monitored in some way, the feedback might not be shared. This applies to everything in healthcare; we record extra information, make codes, but so what?” FG5_C3* *“There is an overwhelming amount of information available on the intranet and hundreds of guidelines depending on the disease group, it becomes challenging to know which ones to follow. When changes are made, staff are being informed, but after many staff members have changed, the new ones are unaware of these updates. This is a significant issue in primary healthcare compared with specialised care where the scope is much narrower, but we have to cover everything.” FG4_C4*
Professional hierarchies Silos Cooperation Influencing Vagueness Uncertainty	**Motivation challenges:** No clear rationales for the value and importance of CPGs provided Lack of shared goals Trust in the agency of individuals	*”I believe the motivation [to read the emailed guidelines] is quite limited. There needs to be a clear understanding of why it’s important for everyone to follow the same process, rather than each person doing what they think is best. This might be the reason why the guidelines are not put into practice” FG2_C1* *“I have a feeling that physicians think that it is your own responsibility to follow evidence-based practice. Although, certainly it would also organization’s responsibility to support evidence-based practices.” FG7_M4*

(FG stands for focus group number, and C/M for clinician/manager)

### Management mechanisms for CPG implementation

The obstacles in managing CPGs highlighted the need for better dissemination strategies, increased motivation, engagement and more manageable information flow to support effective knowledge translation processes. The open-ended questions also allowed interviewees to reveal that, while certain structures and processes existed, they were not necessarily perceived as management tools for implementing CPGs. The interviewees may thus not have even considered that CPG implementation could involve managerial efforts. The identified obstacles reveal problems arising from inadequate managerial practices. Managers and clinicians might not always view CPG implementation as an active management process or collective effort that requires organizational support and interaction. Therefore, to explain how knowledge translation actually occurs, we examined the mechanisms, those critical elements and actions at the organizational level that support the management of CPG implementation. The identified management mechanisms describe the managerial practices and processes through which CPG implementation works.

#### Instructions and process descriptions

Managers reported that the clinical practice advancement and implementation processes of CPGs were organised in various ways, even within a single organization. For example, one had a development group responsible for implementing CPGs across the entire organization. Their decisions were disseminated via email by a chief medical officer for education. Chief physicians, as respected among their peers, were expected to promote the dissemination and acceptance of new instructions. Managers emphasised that all documents were filed in an organization’s databank for continuous access. Supervisors at the work units were considered responsible for disseminating these instructions further, thus managing CPG implementation in clinical practice. Work units could also have their own specific instructions.

#### Accountability structures

Managers highlighted CPG-specific implementation processes and gatherings for important CPG updates, such as for the treatment of blood pressure or diabetes. A smaller group prepared instructions, which were then emailed to all clinicians. According to managers, a chief medical officer for education or clinicians specialised in certain patient groups was responsible for knowledge translation by informing and training staff about new CPGs. It is also noteworthy that managers perceived chief physicians as playing a collective role in advancing clinical practices. Meetings focused primarily on running the practice but occasionally outlined instructions for clinical practice. After managers made a decision, the next step was to inform the staff, leaving the implementation to unit managers, supervisors and clinicians in the work units.

The manager interview data indicate that efforts to advance clinical practices are sometimes detached from formal managerial processes. While interaction is generally expected to occur within units or teams, any policy changes require approval from higher management. Work teams may initiate development or propose changes, but final decisions rest with their superiors. This flexible practice can also cause problems, particularly when attempting to implement changes across the organization. Lack of intraorganizational processes, shared goals, consistent managerial practices and supportive culture makes implementation difficult.

#### Motivation, goal setting and feedback

Despite challenges in accountability structures, the data reveal a growing motivation among staff to influence and improve clinical practices. This pro-active drive reflects clinicians’ desire and commitment to development of care practices, even in contexts where formal opportunities for change are limited. A key managerial practice supporting this motivation is the discussing shared goals within teams and across professional boundaries. These shared objectives help align individual efforts with collective aspirations for better care outcomes, reinforcing a sense of purpose and value of CPGs. Goal setting was particular important for fostering engagement, as it provided clarity and framework for common action. Furthermore, continuous feedback – both formal and informal – was recognised as essential for sustaining motivation, engagement, refining practices, and learning-oriented culture.

#### Communication strategies

Clinicians perceived that work units had gatherings and meetings that provided key communication forums for reviewing and disseminating new CPGs. When instructions for implementing CPGs came from management, clinicians either briefly reviewed them together or were reminded of their receipt. Major changes or those involving multiple professions were typically addressed in dedicated CPG workshops which facilitated cross-disciplinary dialogue and ensured clear, coordinated communication. Face-to-face contact and informal encounters were important mechanisms of action supporting the implementation of CPGs. Clinicians frequently mentioned that education is key for CPG implementation, especially for the deimplementation of low-value care. They actively shared this knowledge with colleagues, fostering a culture of continuous learning and open communication. For example, one unit regularly discussed training and educational content during their weekly meetings, using these sessions as platforms for dialogue and collective understanding.

#### Participatory practices

Importantly, not all changes in clinical practice are driven by managerial decisions or instructions. The clinicians felt that they had good opportunities to initiate changes. Particularly in small organizations, clinicians played a significant role in deciding how to implement CPGs, preparing instructions, creating process descriptions and handling them in multi-professional groups. Clinicians considered chief physicians’ decisions crucial for standardising clinical practices when there are differing opinions. At the unit level, decisions about changing practices were made collectively during meetings, with minimal involvement from management. A common way to propose a change was to contact the nearest chief physician, although it was sometimes unclear who would make the final decision.

The data indicate that chief physicians are key leaders in ensuring that new guidelines are translated, accepted and implemented effectively within primary care organizations. Table 3 summarises the analysis of five key management mechanisms for effective CPG implementation. Mechanisms encompass managerial practices and knowledge translation processes that facilitate CPG implementation. They describe critical elements for leading implementation effectively. Many of the mechanisms also responded to the obstacles identified by the interviewees.

**Table 3:** Management mechanisms for CPG implementation

Identified management mechanism	Quote
**Instructions and process descriptions: **managers play a major role in disseminating instructions for new CPGs, maintaining continuous access to this information and ensuring that all clinicians are informed and understand the guidelines	*“Implementations of CPGs, such as treatment for sleep apnea, or diabetes. We always discuss these with the people involved in the care, then create a process description, and present it in multi-professional workplace meetings to show how we will proceed in the future. Well, process diagrams are forgotten after uploaded to the intranet, but they can be found.”*** FG4_C1**
**Accountability structures: **defining clear responsibilities and accountability supports the implementation of changes in clinical practices and facilitates bottom-up initiatives, allowing clinicians to contribute to the process	*“Although we have CPGs and we make instructions for the whole city, it is still each work unit that decides how to implement them”*** FG6_M6** *“I believe there are about six steps to reach the highest decision-making level, which is the health board. There’s a significant distance in the hierarchy, especially regarding major policies. For minor guidelines, many decisions seem to be made above our unit, limiting our participation. At our health station, we can only make minor adjustments, although this is done collaboratively in a multi-professional manner with GPs, nurses, and other stakeholders involved in the care process, it often feels like we are only able to implement modifications. The rest is decided far away.”*** FG5_C3**
**Motivation, goal setting and feedback:** Management should motivate CPG implementation and provide feedback to both unit managers and clinicians, highlighting shared goals, as well as any challenges or successes in the implementation process	*“Despite having great ideas and people eager to develop things, like those from the intensive care unit– progress often halts. This frustration is a significant issue because moving forward wouldn’t require much time or miracles, just some collective reflection and action.”* **FG1_C1** *“It would be beneficial to gather data and maybe this information could motivate staff if they saw the number of unnecessary or duplicate tests that have been ordered.”*** FG2_C4**
**Communication strategies:** Acting as a communication bridge between operative management and clinicians, managers ensure interaction, dialogue and information flow throughout the organization	*“In the Best Practices group, where we make minor changes to running a practice, we’ve agreed that the chief medical officer for education will implement new CPGs via email. These are usually smaller-scale changes. It’s important to have someone with a strong personal brand, whose emails are opened and who is respected by all professional groups. We’ve received positive feedback about this approach”* **FG6_M2** *“Of course, we try to cover these in our morning meetings. However, the health station with three different locations, approximately 50 GPs, departments and home care in various places, it is so fragmented that it is difficult to provide comprehensive information without using email. While single emails are sent, if there were a clear, effective method for communication, it would likely already be in use*.**” FG3_C7**
**Participatory practices:** Engaging clinicians promotes acceptance and adherence to new guidelines, fostering participatory processes and a collaborative environment, thus making implementation more effective	*“I feel that if something needs to change, a single GP can initiate that change. We listen to ideas and, if necessary, start advancing it. While I can’t speak for other professional groups, GPs can certainly be involved in implementing changes.”* **FG3_C8** *“We have weekly meetings where all the GPs gather, and we also lunch together. This facilitates information sharing among the GPs. Additionally, we hold a monthly department meeting that includes both nurses and GPs at the health station. I would say that information is transferred more effectively face-to-face than via Teams.”*** FG1_C2**

(FG stands for focus group number, and C/M for clinician/manager)

## Discussion

While previous research has focused on strategies for CPG implementation, the managerial aspects in facilitating organizational knowledge translation have been underrepresented. Our findings highlight two diverse perspectives on managing CPG implementation. The main obstacles to managing implementation – dissemination difficulties, motivation challenges and information overload – stem from varying expectations regarding the implementation process. These barriers are well in line with prior implementation studies [7, 17, 18]. However, our study adds that managers view CPG implementation as a matter of resource allocation and one-way information delivery to clinicians. In managing implementation, they saw their role rather indirectly and passively, involving monitoring and providing guidance. Yet, they portrayed the implementation process under someone else responsibility and control, typically by chief physicians.

Conversely, at the operative level, clinicians feel that decisions regarding CPG implementation are made distantly. They expressed a desire to participate in advancing clinical practices but lacked the necessary resources for collective reflection, negotiation or decision-making on how to manage implementation processes together. Clinicians perceived CPG implementation as falling under their expertise and autonomy, with limited involvement from organizational management. In summary, the current focus on knowledge transfer rather than interaction has hindered the translation of new CPGs into shared clinical practice.

This disconnection between implementation perspectives creates a gap that mediates the obstacles to managing CPG implementation. While managers believe that the process is managed successfully at the operational level, clinicians call for more collective decision-making structures or forums to interact to better manage knowledge translation throughout the organization. This finding is important, as prior research on CPG implementation has often overlooked managerial aspects, even though leadership is known to foster complex implementation interventions and a culture of improvement [37]. The literature has identified various barriers and facilitators to translating knowledge in healthcare organizations, listing push and pull factors without problematising a complex link between knowledge and practice [34]. Our study complements this body of work by conceptualising the CPG implementation process from the perspective of those managing knowledge translation and care quality. Our findings shed light on the managerial practices through which CPG implementation can be managed more systematically. We argue that organizational knowledge translation is a social and interactive process, not merely an individual activity or communication. Therefore, human interaction becomes crucial for turning guidelines into shared practices. Organizations with engaged cultures, implementation support and proactive leadership are generally more supportive of evidence-based practices [11].

### Management mechanisms supporting effective implementation

Our study enhances understanding of CPG implementation in primary care by identifying five management mechanisms that promote interaction between managers and clinicians, enabling effective knowledge translation. The findings highlight critical, yet often limited, role managers play in supporting evidence-based practices. In Finnish primary care, the leadership dynamics of multiprofessional teams influence how guidelines are disseminated and integrated into everyday practices. The proposed mechanisms emphasise manager–clinician interaction in fostering collective knowledge for CPG implementation. This aligns with prior studies on middle managers as key knowledge brokers [13, 25, 26] who coordinate implementation, communicate and support professional dialogue. Our findings extend this by showing management mechanisms that operationalise knowledge translation in primary care.

Clinicians in our study raised concerns about the implementation of CPGs in primary care, often misaligned with the limited resources of primary care and organizing follow-up care. Guideline development was perceived rather specialist-driven lacking sufficient contextualisation for primary care settings. This highlights a gap in the relevance and realities of CPG implementation in everyday management, underscoring the need for more interactive and context-sensitive development processes. Managerial practices can play a critical role in bringing this gap by supporting local tailoring, resource alignment, and interprofessional dialogue.

Our results show that CPGs are managed in various ways in primary care organizations. The findings confirm that CPG implementation often occurs in silos, relying on simplistic dissemination rather than active management [33]. Elaborating on knowledge translation models, our study offers concrete ways to adopt a more interactive management approach to motivate and engage clinicians in implementing CPGs. The study contributes to current knowledge on the role of managers in implementing CPGs in primary care. In line with systems thinking, effective CPG implementation in complex care settings requires interactive clinical management practices, not just top-down leadership. It demands organizational policies that empower both top and middle managers in knowledge translation processes [10, 13, 23]. Our proposed management mechanisms contribute to this by offering practical tools that promote leadership, education, stakeholder involvement, and system-level coordination. These mechanisms support holistic quality improvement and risk management [8, 38] helping healthcare organizations and policymakers design more interactive and context-sensitive implementation strategies. Our findings advance the broader discourse on knowledge translation by showing how active management, interaction and clinician dialogue help bridge the know-do gap in primary care.

Managers play a dual role in CPG implementation: as decision-makers in quality management efforts and facilitators of clinical knowledge. Their active involvement ensures that CPGs are not only disseminated but meaningfully integrated into practice, especially in resource-constrained primary care, where clinical complexity is high [5, 18, 31, 39]. Our management mechanisms jointly highlight that successful CPG implementation depends on continuous activation and collective knowledge, supported by communication with frontline staff [14, 16, 40] stakeholder engagement [25, 39], organizational learning [22] and dialogue [33]. These elements of social knowledge formation, enabled by professional interactions and managerial support, are critical management aspects for translating CPGs into everyday practices. They also reflect the culture of improvement [37] and show how organizational practices facilitate the social processes of knowledge formation [41].

In our analysis, there are several ways in which management mechanisms can support CPG implementation. First, organizational instructions and process descriptions provide guidelines for CPG implementation to facilitate communication, dialogue and social knowledge formation among managers and clinicians. These documents should be adapted to the local context to be effective, raising the question of whether CPGs are individual or collective tools. As CPGs are recommended to be implemented nationally, a collective understanding and adoption for use in local clinical practice would be optimal. Organizational instructions and processes support the sharing of knowledge within organizations [33] and facilitate collaboration in linking available knowledge into practice [22].

Second, accountability structures define the roles and responsibilities of the actors involved in the implementation process, ensuring that everyone is accountable for their contributions. By clearly defining expectations and opportunities to influence the process, accountability structures align efforts towards common goals, enhancing commitment, collective knowledge formation and overall effective management of CPG implementation within an organization – also from the bottom up. Our findings highlight the need for follow-up mechanisms to motivate change and ensure shared practices.

Third, as the objective of CPGs is to secure evidence-based practices and quality of care, clinicians require explicit collectively shared goal settings, motivation and performance feedback. Our findings indicate that clinicians are receptive to such feedback. This management mechanism, while common in many other fields, can challenge traditional mechanistic structures in healthcare, such as strong clinician autonomy. Performance-focused cultures and linear interventions often prioritise technical processes over social and behavioural aspects [42], overlooking socially constructed “knowledge in practice” [43–45]. Collective knowledge sharing is known to enhance CPG implementation [15], guideline adherence [19] and intrinsic motivation [46]. Effective management of CPG implementation requires managers to focus on goal-setting, motivation and feedback, supported by performance management systems and interactive forums. The proposed management mechanisms overlap with participatory practices, where reciprocal interaction fosters multi-professional dialogue and transforms guidelines into shared clinical norms. Managers play a key role in leading these discussions, enabling locally tailored implementation strategies and collective knowledge translation in which clinicians can negotiate interpretations and reach consensus on CPGs. These shared understandings form the basis of what is implemented as shared clinical practice.

### Implications

Our study introduces management mechanisms at the organizational level to improve the understanding of the complex system of CPG implementation to provide high-quality care. As managerial practices for CPGs are often inadequate, the mechanisms explain why improvement efforts do or do not work. The key research implication is that management mechanisms support integration and interaction between the spheres of management and clinical practice. While previous studies have focused more on implementation strategies and leadership, this study enhances the understanding of how CPGs can be better managed in practice. Contributing to implementation science, our qualitative study provides valuable insights into developing effective management practices to support CPG implementation in primary care organizations. The results illustrate the obstacles to managing implementation and how new knowledge is assumed to be translated into shared clinical practices, highlighting the complexities of current management efforts for CPG implementation. Instead of viewing knowledge translation as a single dissemination activity or purely an administrative task, we argue that the management of CPG implementation needs collaboration among managers and clinicians to support the social knowledge translation process at the organizational level. Future studies could elaborate on the management mechanisms in other country contexts or clinical settings or in microlevel interactional processes.

These findings have important implications for primary care, particularly for guideline developers and chief physicians. Our findings highlight a need for more inclusive and context-sensitive guideline development processes that reflect the realities of primary care. When all professions are engaged and understand what needs to change and why, CPGs are more effectively communicated and adopted across the care chain, improving care quality and patient safety. For managers, the study calls for the development of organizational knowledge structures that enable and support CPG translation into practice. At the national policy level, such processes could inform the development and dissemination of CPGs across the healthcare system [47]. Overall, the proposed mechanisms offer practical tools to integrate management and implementation strategies, strengthening evidence-based practices in primary care.

### Strengths and limitations

This study addresses the gap in understanding the management of CPG implementation in healthcare organizations. Although the interviews were conducted in Finnish public primary care, it is likely that healthcare organizations, managers and clinicians in other countries face similar implementation challenges. CPGs are targeted at individual clinicians but are also used for planning safer care paths and ensuring equal quality of services. Thus, all healthcare settings must address how to manage CPG implementation effectively. A potential limitation of this study is that focus group discussions might have differed if managers and clinicians had participated together but this might also have inhibited open sharing of practices due to hierarchical dynamics.

## Conclusions

We propose that knowledge translation in healthcare organizations should be managed as a social knowledge formation process rather than a one-way transfer of instructions. This approach allows policymakers to develop practices and structures that support social processes where clinicians can interpret and discuss new CPGs to inform shared clinical practices. Viewing CPGs as collective tools rather than individual responsibilities helps address the implementation obstacles identified by clinicians. This shift also necessitates rethinking the purpose of CPGs. While often perceived as tools for individual clinical decision-making, quality management of care involves multi-actor cooperation. To ensure effective, high-quality and safe patient care requires commitment from all professionals to adhere to shared clinical practices.

## Supplementary Information

Supplementary Material 1.

Supplementary Material 2.

## Data Availability

Interview datasets supporting the results reported in the article are not publicly available due to ongoing nature of the research project but are available from the authors on reasonable request. Upon completion of the project, the anonymized data will be available from Finnish Social Science Data Archive although certain restrictions may apply.

## Abbreviation

CPGs: Clinical practice guidelines
