# Supplementary material for: Implementing clinical practice guidelines into action: a qualitative study of managing knowledge translation in primary care organisations
Source: Health Res Policy Syst. 2025 Oct 14;23:130. doi: 10.1186/s12961-025-01402-z (PMC12522609; doi:10.1186/s12961-025-01402-z)
Supplement: Supplementary file 2 — Supplementary Material 2. [file 12961_2025_1402_MOESM2_ESM.docx]

**Supplement Interview guides**

**Managers’ focus groups**

**Introduction**

The aim of this interview and discussion is to gain a perceptions and understanding how CPGs are implemented and managed. What initiates these changes, what actually happens then. We have two themes, the first one deals with the process, if there is evaluation, where and by whom the decisions are made. The second one concerns the information and knowledge used in changing clinical practice, for example what kind of information is used to argument for the change.

Our objective is to understand the decision making in your work and organization, how Clinical practice guidelines and Choosing wisely recommendations are handled and implemented.

1. What initiates the need to change clinical practice? How does the idea or need for change proceed?

- Do you have practices in work units and in the whole organization to share and implement knowledge (i.e CPGs)
- Once you outline the clinical practice, how do you support the implementation? For example, is somebody responsible for following the implementation?

1. What kind of data, information or knowledge do you have and use

- While you initiate change
- When you argument or motivate for the change
- Follow up implementation

Does your regular reporting system support your decision making, what would you need that you yet do not have?

1. Does the process for de-implementation of low value care [Choosing Wisely recommendations] differ from CPG implementation?

**Clinicians’ focus groups**

**Introduction**

This focus group interview is part of a wider project which aims to promote the use of economic information, evidence on effectiveness and patient provided information in shared decision making. This interview and discussion concentrate on CPGs and if and how economic information could be integrated in CPGs. In addition, we discuss the de-implementation of low value care.

1. **How decision to change clinical practice is made in your workplace?**

- For example, a change in referral practice or instructions on laboratory or imaging services
- How the changes in clinical practice are implemented, put into operation? (Asked only if needed.)
- How do you yourself get information on changes in clinical practice at your workplace? (Asked only if needed.)

1. **What impacts your own clinical practice and treatment decisions?**

- Do economic considerations have an impact on them?
- Why?/Why not?
- In what kind of treatment choices or situations economic considerations have an impact on your decisions?

1. **At your workplace, a decision is made to de-implement low value care. If this happens, how this decision is made?**

- Does the decision making differ from implementing a new practice?
- What raises the need to de-implement practice?

1. **What kind of thought raises the idea to integrate economic information into clinical practice guidelines?**

- Why economic information should be integrated into CPGs? Or why should not it be integrated?
- Do you see any problems in this?
- What kind of economic information should be integrated in CPGs?
  - Could it be
    - Cost per patient per treatment?
    - Costs per all patients having a specific disease?
    - Cost effectiveness of treatment?
  - From whose point of view should the costs be calculated (“who is the payer”)? For example, patient, health care, society?
  - How should the economic information be presented?
    - Text, figures, symbols
    - Comparative tables or figures
    - Treatment order by costs or cost effectiveness?
